# High expression of miR-181c as a predictive marker of recurrence in stage II colorectal cancer

**DOI:** 10.18632/oncotarget.14344

**Published:** 2016-12-28

**Authors:** Nobuyoshi Yamazaki, Yoshikatsu Koga, Hirokazu Taniguchi, Motohiro Kojima, Yukihide Kanemitsu, Norio Saito, Yasuhiro Matsumura

**Affiliations:** ^1^ Division of Developmental Therapeutics, Exploratory Oncology Research & Clinical Trial Center, National Cancer Center, Kashiwa, Japan; ^2^ Department of Colorectal Surgery, National Cancer Center Hospital East, Kashiwa, Japan; ^3^ Pathology Division, National Cancer Center Hospital, Tokyo, Japan; ^4^ Pathology Division, National Cancer Center Hospital East, Kashiwa, Japan; ^5^ Colorectal Surgery Division, National Cancer Center Hospital, Tokyo, Japan

**Keywords:** colorectal cancer, miR-181c, tissue microRNA, predictor of recurrence

## Abstract

**INTRODUCTION:**

A standard treatment for stage II colorectal cancer (CRC) is surgical resection without adjuvant chemotherapy. However, the recurrence rate of these patients is approximately 20%. To date, there are no robust biomarkers suitable for predicting recurrence in stage II CRC patients. In this study, microRNAs (miRNAs) extracted from CRC tissues were examined for a possible biomarker to predict recurrence in stage II CRC patients.

**RESULTS:**

From the comprehensive analysis, 15 miRNAs were selected as candidates for further study. Regarding let-7a, -7d, -7e, miR-23c, -26b, -128a, -151-5p, and -181c, recurrence rates in training cohort patients with higher expression of these miRNAs isolated from their frozen tissues samples were significantly higher than those with lower expression (*P* < 0.05). According to multivariate analysis, the higher expression of miR-181c was detected as an independent predictive factor of recurrence (*P* = 0.001, OR: 9.43, 95% CI: 2.57–34.48). Results were similar in miR-181c extracted from FFPE tissues obtained from the training cohort (*P* = 0.003, OR: 7.46, 95% CI: 1.97–28.57). In the validation cohort using FFPE tissues, the recurrence rate in patients with higher miR-181c expression was significantly higher than those with lower miR-181c expression (*P* < 0.001).

**MATERIAL AND METHODS:**

Comprehensive analysis using a highly sensitive miRNA chip was initially performed to select candidate miRNAs associated with recurrence. Candidate miRNAs were analyzed by real-time RT-PCR using RNA from frozen and formalin-fixed, paraffin-embedded (FFPE) tissues.

**CONCLUSIONS:**

Higher expression of miR-181c may be a useful recurrence predictor of stage II CRC patients.

## INTRODUCTION

Colorectal cancer (CRC) is the second leading cause of worldwide cancer-related mortality [1]. The standard treatment for patients with CRC from stages I to III is surgical resection. Stage II CRC was defined as that the tumor invades deeply through the muscularis propria without lymph node metastasis and distant metastasis, and Stage III CRC was defined as that the tumor invades deeply through the muscularis propria with lymph node metastasis and without distant metastasis. The effectiveness of adjuvant chemotherapy for stage III CRC patients has been shown [2, 3]. On the other hand, the effectiveness of adjuvant chemotherapy for stage II CRC patients has not been established to date. However, the recurrence rates of stage II CRC patients who underwent surgery alone were about 12%–37% [4–8]. Therefore, adjuvant chemotherapy for high-risk stage II patients could be considered in the guidelines of American Society of Clinical Oncology [9] and European Society for Medical Oncology [10]. According to the guidelines, the high-risk stage II group of CRC patients was defined by the clinicopathological factors classified as follows, pathological T4 stage; it means that the tumor perforates the peritoneum or invades other organs according to the UICC 7th TNM classification, <12 analyzed lymph nodes, vascular or lymphatic or perineural invasion, poorly differentiated histology, and clinical manifestation with intestinal occlusion or perforation. However, there were no robust biomarkers suitable for detecting recurrence in stage II CRC patients. To select stage II CRC patients suitable for adjuvant chemotherapy, the investigation for the adequate biomarkers is considered to be important for clinical settings.

MicroRNAs (miRNAs), which are small (18–25 nucleotides in size) noncoding RNA molecules, are known to regulate the function of specific mRNAs and play various roles in cancer progression. The function of miRNAs is to downregulate the expression of multiple target genes by degrading their corresponding mRNAs or blocking gene expression and subsequent translation into protein via RNA interference [11, 12]. Totally, 2661 human mature miRNAs have been bioinformatically reported in miRBase 21 in June 2014 [13]. A recent study has clarified that circulating miRNAs are remarkably stable in plasma due to their resistance to endogenous RNase activity [14]. Recent studies have demonstrated that several miRNAs play important roles in tumor invasion and metastasis [15, 16] and have clarified that several miRNAs in tissue samples are potential biomarkers for CRC recurrence [17, 18]. Thus, miRNAs seems to be worthy of investigation as tumor biomarkers for CRC recurrence.

In the present study, miRNAs extracted from the tissue samples of stage II CRC patients who had undergone surgical resections were assessed as biomarkers to predict the recurrence of stage II CRC.

## RESULTS

### Study participants

Table 1 shows the characteristics of the patients enrolled in this study. In the preliminary study, there were no significant differences between the stage II CRC patients with and without recurrence regarding the clinicopathological factors. In the training cohort, 14 patients had recurrence, whereas 66 patients were recurrence-free. In contrast to recurrence-free CRC patients, the number of CRC patients with recurrence had significantly higher T4 tumor depths, which was also seen in the histology samples from mucinous carcinoma (*P* < 0.05). In the validation cohort, 6 patients were excluded because of poor miRNA quality. Thus, 57 patients were analyzed. Seven patients showed recurrence and 50 patients were recurrence-free. In those patients, the recurrence rate was significantly higher in rectal cancer than in colon cancer (*P* < 0.05).

**Table 1:** Charactaristics in each study

Characteristics	Preliminary study			Training cohort			Validation cohort		
	Patients with recurrencen=5	Patients without recurrencen=5	*P* value	Patients with recurrencen=14	Patients without recurrencen=66	*P* value	Patients with recurrencen=7	Patients without recurrencen=50	*P* value
Age, y			0.402			0.718			0.519
Median	65	68		62	63		66	67	
Range	58 — 78	60 — 71		32 — 78	38 — 83		40 — 76	43 — 85	
Sex, no. (%)			1			0.730			0.421
Male	4 (80)	4 (80)		10 (71.4)	44 (66.7)		4 (57.1)	36 (72.0)	
Female	1 (20)	1 (20)		4 (28.6)	22 (33.3)		3 (42.9)	14 (28.0)	
Tumor location, no. (%)			0.490			0.400			0.006
Colon	3 (60)	4 (80)		7 (50.0)	41 (62.1)		0 (0)	28 (56.0)	
Rectum	2 (40)	1 (20)		7 (50.0)	25 (37.9)		7 (100)	22 (44.0)	
Tumor size, mm			0.753			0.563			0.990
Median	55	50		45	45		50	50	
Range	30-74	35-70		15-140	20-90		28-70	20-105	
Tumor depth, no (%)			1			0.048			0.440
T3	5 (100)	5 (100)		9 (64.3)	57 (86.4)		6 (85.7)	36 (7.0)	
T4	0	0		5 (35.7)	9 (13.6)		1 (14.3)	14 (28.0)	
Histology, no. (%)			1			0.048			0.438
W/D and M/D	5 (100)	5 (100)		12 (85.7)	65 (98.5)		7 (100)	46 (92.0)	
P/D and Muc	0	0		2 (14.3)	1 (1.5)		0 (0)	4 (8.0)	
Lymphatic invasion, no. (%)			1			0.956			0.076
Positive	1 (20)	1 (20)		1 (7.1)	5 (7.6)		1 (14.3)	25 (50.0)	
Negative	4 (80)	4 (80)		13 (92.9)	61 (92.4)		6 (85.7)	25 (50.0)	
Venous invasion, no. (%)			1			0.837			0.440
Positive	2 (40)	2 (40)		7 (50.0)	31 (47.0)		6 (85.7)	36 (72.0)	
Negative	3 (60)	3 (60)		7 (50.0)	35 (53.0)		1 (14.3)	14 (28.0)	
Observation period, months			0.602			0.723			< 0.001
Median	107	74		70	75		48	83	
Range	25 — 127	61 — 86		25 — 127	40 — 127		35 — 65	18 — 91	

CRC: colorectal cancer, W/D: well-differentiated adenocarcinoma, M/D: moderately differentiated adenocarcinoma, P/D: poorly differentiated adenocarcinoma, Muc: mucinous carcinoma, RFS: relapse free survival, The differences were analyzed by Mann-Whitney U test, chi-square, or log-rank test. P < 0.05 denotes statistically significant difference.

### Preliminary study

Three tumor tissue specimens with recurrence were excluded from the analysis because of poor miRNA quality. Thus, seven tumor specimens, which were two with recurrence and five without recurrence, and ten normal specimens were analyzed.

**Figure 1:**
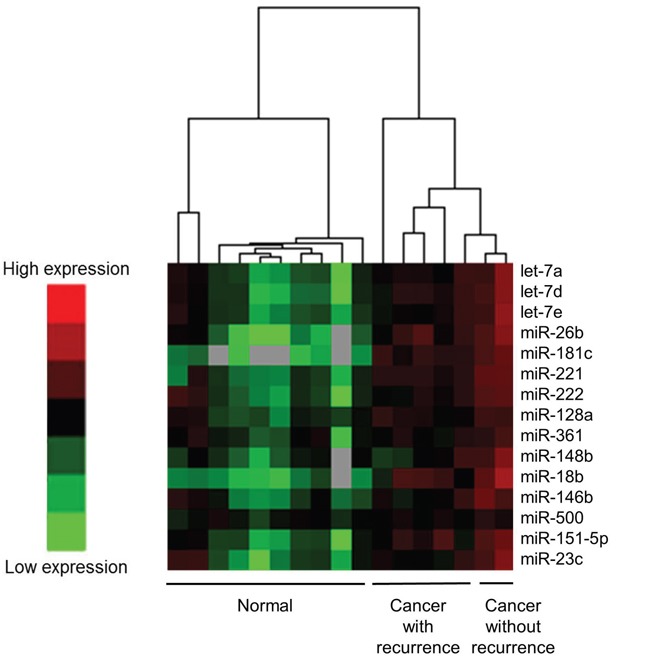
Differences in miRNA expression in the frozen tissues between cancer tissues with recurrence and those without recurrence using a heat map for the preliminary study A total of 15 species of miRNAs showed significantly higher expression in cancer tissue than in noncancerous tissues; in addition, miRNAs from cancer tissues with recurrence rather than from cancer tissues without recurrence were selected. miRNAs were let-7a, -7d, -7e, miR-18b, -23c, -26b, -128a, -146b, -148b, -151-5p, -181c, -221, -222, -361, and -500.

Initially, miRNAs which were expressed higher in the cancer tissues rather than those in the normal tissues were selected. In 1719 miRNAs, 394 miRNAs showed different expressions of miRNA between cancer and normal tissues (*P* < 0.01). In 394 miRNAs, 105 miRNAs in the cancer tissues showed greater expressions than those in the normal tissues (Supplementary Figure 1). Among the selected miRNAs which were expressed higher expressions in the cancer tissues of the patients with recurrence than those without recurrence were obtained. Fifteen of 105 miRNAs, including let-7a, -7d, -7e, miR-18b, -23c, -26b, -128a, -146b, -148b, -151-5p, -181c, -221, -222, -361, and -500 showed significantly higher expressions in cancer tissues from patients with recurrence than in cancer tissues from those without recurrence (*P* < 0.05) (Figure 1).

### miRNA expressions in the frozen cancer tissues

For miRNA expression analysis, 15 candidate miRNAs selected from the highly sensitive miRNA array were analyzed using U6 as internal control. Supplementary Table 1 shows Ct values, and Table 2 shows RQ obtained by real-time PCR for the candidate miRNAs from the frozen tissues. In almost all samples, Ct values could be measured. However, Ct values of miR-18b could not be determined in 30 samples. Thus, miR-18b was excluded from further analysis. Using ROC curves with the Youden index, the threshold and AUC of each miRNA was established (Table 2 and Supplementary Figure 2A). In addition, miRNAs with AUC < 0.6 were excluded from further analysis. Higher expression was defined as equal or higher miRNA expression than the threshold expression. Lower expression was defined as lower expression than threshold expression.

**Table 2:** Relative quantification of miRNA normalized to U6snRNA in the frozen tissue and FFPE tissue of training cohort

	CRC patients with recurrence, n=14		CRC patients without recurrence, n=66		Threshold	AUC
	median	range	median	range		
Frozen Tissue						
let-7a	0.16	0.051 – 0.40	0.095	0.011 – 0.55	0.181	0.680
let-7d	0.026	0.0081 – 0.069	0.015	0.0024 – 0.11	0.014	0.698
let-7e	0.26	0.072 – 0.78	0.19	0.034 – 1.24	0.451	0.672
miR-18b	1.7 × 10^-5^	0 – 4.5 × 10^-4^	4.6 × 10^-6^	0 – 1.3 × 10^-4^	2.7 × 10^-5^	0.669
miR-23c	0.0025	1.4 × 10^-4^ – 0.0081	9.9 × 10^-4^	8.4 × 10^-5^ – 0.0090	0.002	0.724
miR-26b	0.27	0.031 – 0.74	0.22	0.028 – 1.53	0.496	0.642
miR-128a	0.018	0.0037 – 0.076	0.012	0.0010 – 0.092	0.035	0.632
miR-146b	0.22	0.0096 – 1.21	0.22	0.026 – 0.76	0.116	0.535
miR-148b	0.014	0.0017 – 0.070	0.0075	0.0015 – 0.033	0.007	0.686
miR-151-5p	0.024	0.0027 – 0.17	0.017	0.0020 – 0.085	0.022	0.633
miR-181c	0.0069	8.3 × 10^-4^ – 0.022	0.0042	4.4 × 10^-4^ – 0.032	0.007	0.729
miR-221	0.14	0.045 – 1.08	0.11	0.018 – 0.71	0.444	0.483
miR-222	0.40	0.075 – 1.35	0.48	0.036 – 1.19	0.791	0.462
miR-361	0.013	0.0020 – 0.052	0.0075	8.1 × 10^-4^ – 0.063	0.009	0.682
miR-500	0.0032	5.3 × 10^-4^ – 0.019	0.0036	2.0 × 10^-4^ – 0.020	0.008	0.526
FFPE tissue						
let-7a	0.23	0.096 – 0.55	0.20	0.051 – 0.68	0.245	0.542
let-7d	0.096	0.047 – 0.21	0.75	0.021 – 0.21	0.111	0.658
let-7e	0.31	0.16 – 0.59	0.25	0.083 – 0.63	0.303	0.618
miR-23c	0.012	0.0033 – 0.029	0.012	0.0059 – 0.019	0.023	0.557
miR-26b	0.21	0.10 – 0.43	0.16	0.049 – 0.43	0.174	0.668
miR-128a	0.0090	0.0037 – 0.020	0.0076	0.0024 – 0.026	0.008	0.641
miR-148b	0.0015	0.0059 – 0.019	0.0097	0.0029 – 0.040	0.010	0.675
miR-151-5p	0.024	0.0072 – 0.040	0.017	3.4 × 10^-5^ – 0.057	0.021	0.670
miR-181c	0.0091	0.0041 – 0.015	0.0053	0.0018 – 0.018	0.010	0.694
miR-361	0.0093	0.0027 – 0.016	0.0071	0.0015 – 0.021	0.009	0.617

CRC: colorectal cancer, AUC: area under the curve, The thresholds were established using ROC curve and Youden index.

**Figure 2:**
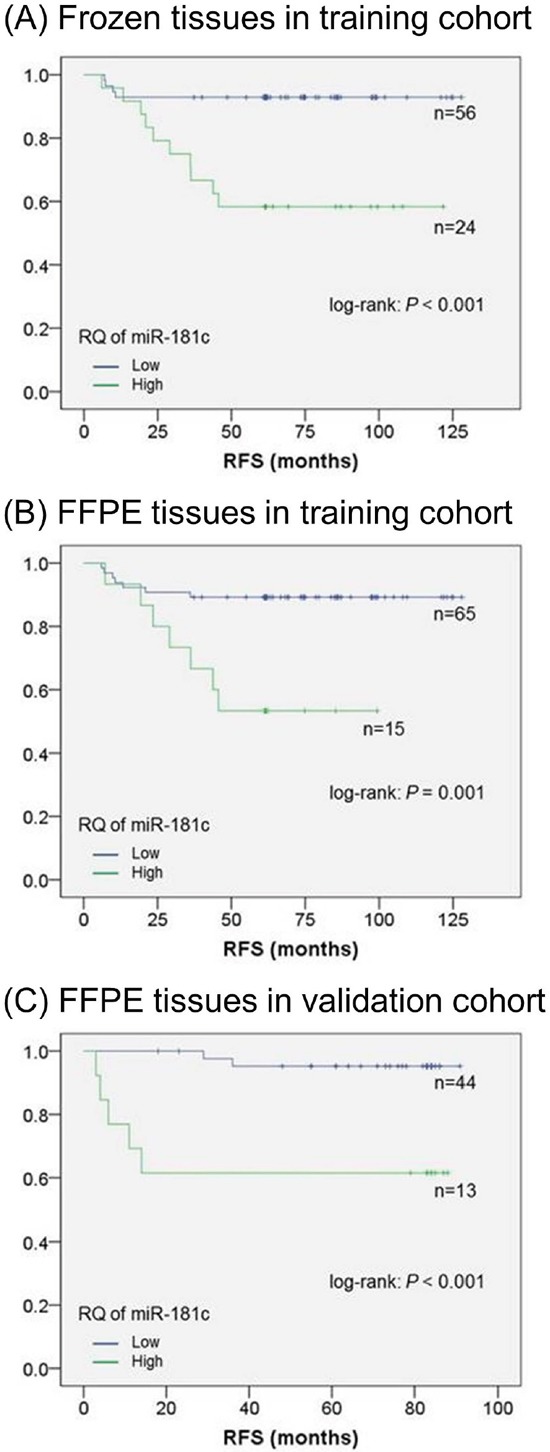
Relapse-free survival of stage II CRC **A**. Kaplan–Meier method for the two groups in the training cohort using frozen tissue that showed both higher and lower miR-181c expressions. The threshold was established using the ROC curve and Youden index. RFS was significantly worse for the patients with higher miR-181c expression than for those with lower miR-181c expression of. **B**. Kaplan–Meier method for the two groups in the training cohort using FFPE tissue that showed both higher and lower miR-181c expressions. The threshold was established using the ROC curve and Youden index. RFS was significantly worse for the patients with higher miR-181c expressions than for those with lower miR-181c expressions. **C**. Kaplan–Meier method for the two groups in the validation cohort that showed both high and low miR-181c expressions. The threshold was 0.010, which was established in the training cohort. RFS was significantly worse for the patients with higher miR-181c expression than for those with lower miR-181c expressions. The differences were analyzed by log-rank test. *P* < 0.05 denotes a statistically significant difference.

Table 3 shows the miRNA expression characteristics as well as pathological factors. Tumor recurrence occurred at significantly higher rates in the patients with a higher expression of let-7a, -7d, -7e, miR-23c, -26b, -128a, -151-5p, -181c, a tumor depth of T4, and histological type of mucinous carcinoma (*P* < 0.05). In a multivariate analysis including pathological factors, the higher expression of miR-181c was an independent predictive factor of recurrence [odds ratio (OR): 9.43, 95% confidence intervals (CI): 2.57–34.48, *P* = 0.001]. RFS was significantly worse in the patients with the higher expression of let-7a, -7d, -7e, miR-23c, -26b, -128a, -181c, and the histological type of mucinous carcinoma (*P* < 0.05). In a multivariate analysis including pathological factors, a higher miR-181c expression was also an independent predictive factor of worse RFS [hazard ratio (HR): 6.62, 95% CI: 2.08–21.28, *P* = 0.001]. Figure 2A shows the RFS curves according to the expressions of miR-181c extracted from frozen tissues obtained from the training cohort.

**Table 3:** miRNA expression in the frozen tissue and pathological characteristics compared with and without recurrence

	Patients with recurrencen=14, (%)		Patients without recurrencen=66, (%)		Chi-square	Multivariate analysis for recurrence			3 yearRFS rate (%)	log-rank	Multivariate analysis for RFS		
					*P* value	Odds ratio	95% C.I.	*P* value		*P* value	Hazard ratio	95% C.I.	*P* value
let-7a					0.045			0.352		0.040			0.367
High (n=18)	6	(42.9)	12	(18.2)					66.7				
Low (n=62)	8	(57.1)	54	(81.8)					90.3				
let-7d					0.006			0.086		0.007			0.103
High (n=48)	13	(92.9)	35	(53.0)					79.2				
Low (n=32)	1	(7.1)	31	(47.0)					96.9				
let-7e					0.003			0.121		0.002			0.167
High (n=13)	6	(42.9)	7	(10.6)					61.5				
Low (n=67)	8	(57.1)	59	(89.4)					91.0				
miR-23c					0.008			0.086		0.009			0.135
High (n=27)	9	(64.3)	18	(27.3)					74.1				
Low (n=53)	5	(35.7)	48	(72.7)					92.5				
miR-26b					0.001			0.171		0.001			0.213
High (n=11)	5	(35.7)	6	(9.1)					55.6				
Low (n=69)	9	(64.3)	60	(90.9)					90.1				
miR-128a					0.001			0.264		0.006			0.216
High (n=11)	5	(35.7)	6	(9.1)					63.4				
Low (n=69)	9	(64.3)	60	(90.9)					88.4				
miR-148b					0.079			0.558		0.082			0.490
High (n=46)	11	(78.6)	35	(53.0)					80.4				
Low (n=34)	3	(21.4)	31	(47.0)					94.1				
miR-151-5p					0.012			0.883		0.118			0.780
High (n=31)	8	(57.1)	23	(34.9)					77.4				
Low (n=49)	6	(42.9)	43	(65.1)					89.8				
miR-181c					< 0.001	9.43	2.57 – 34.48	0.001		< 0.001	6.62	2.08 – 21.28	0.001
High (n=24)	10	(71.4)	14	(21.2)					70.8				
Low (n=56)	4	(28.6)	52	(78.8)					92.9				
miR-361					0.088			0.645		0.094			0.579
High (n=35)	9	(64.3)	26	(39.4)					80.0				
Low (n=45)	5	(35.7)	40	(60.6)					91.1				
Tumor depth					0.048			0.222		0.094			0.3191
T4 (n=14)	5	(35.7)	9	(13.6)					75.0				
T3 (n=66)	9	(64.3)	57	(86.4)					88.2				
Histology					0.022			0.125		0.002			0.105
Muc (n=3)	2	(14.3)	1	(1.5)					33.3				
W/D and M/D (n=77)	12	(85.7)	65	(98.5)					88.3				

C.I.: confidence interval, W/D: well-differentiated adenocarcinoma, M/D: moderately differentiated adenocarcinoma, Muc: mucinous carcinoma, High: higher expression of miRNA than the threshold, Low: equal or lower expression of miRNA than the threshold. The differences were analyzed by chi-square test, log-rank test or Multivariate logistic regression analysis. P < 0.05 denotes statistically significant difference.

### miRNA expressions in the FFPE cancer tissues

According to the results of the frozen tissue study, the candidate miRNA, including let-7a, -7d, -7e, miR-23c, -26b, -128a, -148b, -151-5p, -181c, and -361-5p, were analyzed using FFPE tissues of the same patients in the training cohort. Supplementary Table 1 shows Ct values and Table 2 also shows the RQ, threshold, and AUC of the candidate miRNA of the FFPE tissues (Supplementary Figure 2B). In the analysis using FFPE tissues, miRNAs with AUC < 0.6 were excluded from further analysis. Higher expression and lower expression was defined as well as frozen cancer tissues.

Table 4 shows the characteristics of the miRNA expression profiles as well as pathological factors. Patients with the higher expressions of let-7d, -7e, miR-26b, -128a, -148b, -151-5p, -181c, a tumor depth of T4, and the histological type of mucinous carcinoma (*P* < 0.05) recurred at significantly higher rates. In a multivariate analysis including pathological factors, the higher expression of miR-181c was an independent predictive factor of recurrence (OR: 7.46, 95% CI: 1.97–28.57, *P* = 0.003). RFS was significantly worse in the patients with higher expressions of let-7d, miR-26b, -128a, -148b, -181c, and the histological type of mucinous carcinoma (*P* < 0.05). In a multivariate analysis including pathological factors, the higher expression of miR-181c was also an independent predictive factor worse RFS (HR: 4.74, 95% CI: 1.66–13.51, *P* = 0.001). A statistically positive correlation in miR-181c expression between the frozen and FFPE tissues in these patients (*ρ* = 0.257; *P* = 0.021; Spearman's correlation analysis) (Supplementary Figure 3) was observed. Figure 2B shows the RFS curves according to the expression of miR-181c extracted FFPE tissues obtained from the training cohort.

**Table 4:** miRNA expressions in the FFPE tissue and pathological characteristics compared with and without recurrence

	Patients with recurrencen=14, (%)		Patients without recurrencen=66, (%)		Chi-square	Multivariate analysis for recurrence			3 yearRFS rate (%)	log-rank	Multivariate analysis for RFS		
					*P* value	Odds ratio	95% C.I.	*P* value		*P* value	Hazard ratio	95% C.I.	*P* value
let-7d					0.004			0.249		0.004			0.174
High (n=17)	7	(50.0)	10	(15.2)					70.6				
Low (n=63)	7	(50.0)	56	(84.8)					88.9				
let-7e					0.030			0.505		0.339			0.397
High (n=26)	8	(57.1)	18	(27.3)					76.9				
Low (n=54)	6	(42.9)	48	(72.7)					88.9				
miR-26b					0.038			0.153		0.044			0.283
High (n=37)	10	(71.4)	27	(40.9)					81.1				
Low (n=43)	4	(28.6)	39	(59.1)					90.7				
miR-128a					0.038			0.282		0.0438			0.285
High (n=37)	10	(71.4)	27	(40.9)					81.1				
Low (n=43)	4	(28.6)	39	(59.1)					90.7				
miR-148b					0.006			0.173		0.007			0.079
High (n=42)	12	(85.7)	30	(45.5)					78.6				
Low (n=38)	2	(14.3)	36	(54.5)					94.7				
miR-151-5p					0.012			0.341		0.117			0.252
High (n=28)	9	(64.3)	19	(28.8)					77.2				
Low (n=52)	5	(35.7)	47	(71.2)					89.8				
miR-181c					0.001	7.46	1.97 – 28.57	0.003		0.001	4.74	1.66 – 13.51	0.004
High (n=15)	7	(50.0)	8	(12.1)					66.7				
Low (n=65)	7	(50.0)	58	(87.9)					89.2				
miR-361					0.056			0.694		0.076			0.894
High (n=28)	8	(57.1)	20	(30.3)					79.8				
Low (n=52)	6	(42.9)	46	(69.7)					88.5				
Tumor depth					0.048			0.627		0.094			0.150
T4 (n=14)	5	(35.7)	9	(13.6)					75.0				
T3 (n=66)	9	(64.3)	57	(86.4)					88.2				
Histology					0.022			0.070		0.002			0.066
Muc (n=3)	2	(14.3)	1	(1.5)					33.3				
W/D and M/D (n=77)	12	(85.7)	65	(98.5)					88.3				

C.I.: confidence interval, W/D: well-differentiated adenocarcinoma, M/D: moderately differentiated adenocarcinoma, Muc: mucinous carcinoma, High: higher expression of miRNA than the threshold, Low: equal or lower expression of miRNA than the threshold. The differences were analyzed by chi-square test, log-rank test or Multivariate logistic regression analysis. P < 0.05 denotes statistically significant difference.

### Correlation between the expression of miR-181c and PTEN

To explain the correlation between the expression of miR-181c and PTEN, they were analyzed in the training cohort using frozen tissues. Supplementary Figure 4 shows the correlation obtained by real-time PCR for the miR-181c and PTEN from the frozen tissues. The expression of PTEN was significantly lower in the patients with higher miR-181c expression than those with lower expression (*P* = 0.023).

### Validation study

In the training cohort, miR-181c was considered to be an independent predictive factor of recurrence and RFS in frozen as well as FFPE tissues. Therefore, miR-181c was chosen for further validation and analysis using FFPE tissues of another cohort. The threshold of RQ was defined as 0.010 due to the threshold in the training cohort's FFPE tissues (Table 2 and Supplementary Figure 2B).

According to the Ct values shown in Supplementary Table 1, RQ was established. AUC of RQ for miR-181c was 0.771 (Supplementary Figure 2C). Table 5 shows the relationship between the miR-181c expression and tumor recurrence. Tumor recurrence occurred at a significantly higher rate in patients with higher miR-181c expression than in those with lower expression (*P* = 0.001). Figure 2C shows the RFS curves according to the miR-181c expression in the validation cohort. RFS was significantly worse for the patients with higher miR-181c expression than for those with lower miR-181expression (*P* < 0.001).

**Table 5:** miR-181c expression in the validation study compared with and without recurrence

	Patients with recurrencen=7, (%)		Patients without recurrencen=50, (%)		Chi-square	3 yearRFS rate (%)	log-rank
					*P* value		*P* value
miR-181c					0.001		< 0.001
High (n=13)	5	(71.4)	8	(16.0)		61.5	
Low (n=44)	2	(28.6)	42	(84.0)		95.2	

## DISCUSSION

We previously reported the expression profile of miRNA in fecal samples [19–22]. Recently, several studies have indicated that miRNA extracted from blood samples could be useful for the diagnosis of CRC [23, 24]. In addition, several studies have indicated that miRNAs may be potential biomarkers of either CRC metastases or recurrence [25, 26]. In the present study, we focused on the relationship between the expression of miRNA extracted from CRC tissue samples and its use as a predictive marker of recurrence.

RQ was applied to analyze the expressions of miRNA. Therefore, an appropriate internal control was required to normalize the variation of each sample. However, there was no consensus on the appropriate internal control to evaluate RQ of miRNA. Usually, U6 [19, 27, 28] or RNU6B [29–31] were used as internal controls. In addition, miR-24 was used as an internal control in our previous studies [20–22]. Because RNU6B and miR-24 showed higher variability and larger difference between the patients than U6, U6 was chosen as the internal control in the present study. Although U6 showed high stability in the frozen and FFPE tissues from the training cohort, 6 patients in the validation cohort showed lower expression of U6 probably due to the poor storage conditions. Consequently, 6 patients in the validation cohort with lower expressions of U6 were excluded from the present analysis. The threshold of Ct value for U6 was defined as 24 following the analysis of Ct values from all 223 samples, including frozen and FFPE tissues (data not shown).

In the present study, frozen tissues were initially used for comprehensive analysis and selection of candidate miRNAs. However, it is difficult to obtain frozen tissues and to store them for a long term compared with FFPE tissues. miRNAs is known to be preserved in poor conditions even in FFPE sections stored for 20 years [30]. However, miRNA expressions and thresholds may be altered depending on a storage conditions. Thus, FFPE tissues were analyzed in comparison with their corresponding frozen tissues in the same cohort. The miRNA expression analysis showed similar results between frozen and FFPE tissues from these patients. In addition, a significant positive correlation of miR-181c expression was observed between frozen and FFPE tissues.

To date, there have been numerous reports indicating a strong correlation of miRNA status with CRC. However, there were limited numbers of reports about miRNA expression in stage II CRC. For example, miR-21 [32, 33], miR-29a [18], miR-34a-5p [34], miR-148a [35], and others [36, 37] were reported as predictive factors for recurrence in stage II CRC patients. Recently, six-miRNA-based classifier, which contained miR-21-5p, -20a-5p, -103a-3p, -106b-5p, -143-5p, and -215, had been reported as a prognostic and a predictive tool for disease recurrence in stage II CRC patients [38]. miR-181c was not selected in the study; however, the miRNAs reported by Zhang JX et al. were selected using cancer and adjacent normal mucosa, whereas, the candidate miRNAs in the present study were selected as follows; miRNAs which were expressed higher in the cancer tissues rather than those in the normal tissues were initially selected, and the miRNAs which were expressed higher in the cancer tissues of the patients with recurrence than those without recurrence were finally obtained. Indeed, five of six-miRNAs were selected in the preliminary study (Supplementary Figure 1). Moreover, there were no studies using both frozen and FFPE tissues. It was indicated in the present study that miR-181c isolated from the frozen and FFPE tissues can be an independent predictive factor of recurrence and RFS in stage II CRC patients. Previous studies reported that miR-181c of tissues and plasma from CRC patients showed higher expression than miR-181c from healthy controls [29, 39]. In addition, several studies indicated that the expression of miR-181c was associated with poor prognosis in various cancers. For example, high expressions of miR-181c were observed in gastric cancer patients [40] or in those with early recurrence of glioblastoma [41]. Biochemical studies also showed that miR-181c suppressed phosphatase and tensin homolog gene (PTEN) expression in inflammatory breast cancer tissues by targeting its 3′-UTR and promoting proliferation [42]. Previous studies suggested that the PTEN/phosphoinositide 3-kinase (PI3K)/phosphorylated Akt (pAkt) pathway may play an important role in sporadic colon carcinogenesis [43, 44] and loss of PTEN expression was a predictive marker for a recurrence of stage II CRC patients [44]. Indeed, the expression of PTEN was significantly lower in the patients with higher miR-181c expression than those with lower expression in the present study. It was slight difference in the expression of PTEN between higher and lower miR-181c expression in the present study. However, this result had statistically significant difference. Therefore, high miR-181c expression may be involved in the metastasis of CRC through the PTEN/PI3K/pAkt pathway. Other types of miR-181 family were reported to be associated with CRC. Previous studies reported that miR-181a was upregulated in CRC tissues [39, 45], and miR-181a was upregulated by oncogenic KRAS [46]. Another study reported that miR-181b showed higher expression in cancer tissues of CRC than in noncancerous tissues [31, 39, 47, 48]. Thus, the miR-181 family may play an important role in CRC carcinogenesis.

In the present study using FFPE tissues combined with training and validation cohorts, 12 patients among the 28 patients who showed higher expressions of miR-181c had recurrence. In contrast, 9 patients among the 109 patients who showed lower expressions of miR-181c also had recurrence. The recurrence rates were 42.9% in higher and 8.3% in lower miR-181c expressions, respectively. The recurrence rate in stage II CRC patients with higher expressions of miR-181c was similar to that of stage III CRC patients who had been treated by surgical resection alone [49, 50]. Furthermore, in the patients with lower miR-181c expression, the recurrence rate of the present stage II CRC patients was similar or lower compared with that of stage I CRC patients [51]. Supplementary Figure 5 shows the result compared to 80 patients with stage II CRC patients and 121 patients with stage III CRC patients in the preliminary study using frozen tissues. The expression of miR-181c in stage II CRC patients without recurrence was not showed significantly difference from that in the stage III CRC patients without recurrence (*P* = 0.297). Moreover, that in stage II CRC patients with recurrence was similar compared with that in stage III patients with recurrence (*P* = 0.825). Results from this study indicate that RQ of miR-181c could be a potential biomarker to detect stage II CRC. These findings suggest that stage II CRC patients with higher miR-181c expressions could be candidates for adjuvant chemotherapy.

Because the present data are obtained from the retrospective study conducted at a single institution and had a small number design, prospective studies are needed to evaluate the prognostic significance of miR-181c expression for stage II CRC recurrence.

## MATERIALS AND METHODS

### Study participants

This study was approved by the ethical committee of the National Cancer Center, Japan. All tissue samples were obtained with the informed consent from the patients.

In the preliminary and training cohorts, 304 CRC patients who had undergone surgical resection between January 2003 and December 2009 were enrolled. All patients had undergone surgery at the National Cancer Center Hospital, Tokyo, Japan, and the frozen cancer tissue and adjacent noncancerous portions were stored. Eighty-two patients were classified as having stage II CRC. Two patients were excluded because of comorbid hepatocellular carcinoma and severe necrosis of the sample. Thus, 80 stage II CRC patients were eligible for analysis. None of these patients had received adjuvant chemotherapy.

In the preliminary study, 5 patients with recurrence and 5 patients without recurrence were enrolled because they had similar characteristics to each other. In the training cohort, 80 patients were enrolled, and the expressions of miRNAs in frozen and formalin-fixed, paraffin-embedded (FFPE) tissues were retrospectively analyzed.

In the validation cohort, 287 CRC patients who underwent surgical resection between January 2006 and December 2006 were enrolled. All patients had undergone surgery at the National Cancer Center Hospital East, Kashiwa, Japan. Sixty-nine patients were classified as having stage II CRC, and 6 patients were excluded because no clinicopathological data were available. Thus, 63 stage II CRC patients were eligible for analysis. miRNAs expressions in the FFPE tissues were retrospectively analyzed. None of these patients had received adjuvant chemotherapy.

### Total RNA extracted from frozen tissues

Total RNA was extracted using the miRNeasy Mini Kit (Qiagen, Valencia, CA) according to the manufacturer's instructions with a slight modification. Approximately 50 mg of each frozen tissue sample were put into tubes containing ceramic beads and homogenized with 1 mL of QIAzol (Qiagen), using a Precellys 24 device (Bertin Technologies, Saint-Quentin-en-Yvelines Cedex, France) at 6,500 rpm for 50 s. The mixture was processed according to the manufacturer's instructions. RNA concentrations were measured by a NanoDrop (Thermo Scientific, Wilmington, DE), and the quality of RNA was measured by a 2100 Bioanalyzer (Agilent Technologies, Santa Clara, CA). The RNA samples were stored at −80°C until use.

### Highly sensitive miRNA microarray

First, a comprehensive analysis of miRNAs was performed to select candidate miRNAs. Total RNAs extracted from the frozen cancer tissues and normal tissues were labeled with 3D-Gene miRNA labeling kit (TORAY, Kamakura, Japan). Labeled RNAs were hybridized onto 3D-Gene Human miRNA Oligo chips (TORAY). The annotation and oligonucleotide sequences of the probes were matched to the miRNA database 17 [miRBase ( http://www.mirbase.org)]. After stringent washes, fluorescent signals were scanned with the 3D-Gene Scanner (TORAY) and analyzed using 3D-Gene Extraction software (TORAY).

### cDNA synthesis and miRNA expression analysis by real-time RT-PCR

miRNA extracted from the cancer portions of the frozen tissues from the training cohort patients were analyzed. cDNA was synthesized using a High-Capacity TaqMan MicroRNA RT Kit (Applied Biosystems, Foster, CA) in accordance with the manufacturer's instructions. The reaction mixture consisted of 3 ng of total RNA, 0.5 μL of 10 × RT buffer, 1 μL of 5 × specific primer, 0.05 μL of 25 × dNTPs (100 mM), 0.06 μL of RNase inhibitor (20 U/μL), and 0.33 μL of MultiScribe reverse transcriptase (50 U/μL) in a final reaction volume of 5 μL. The thermal cycling conditions were as follows: 1) 16°C for 30 min; 2) 42°C for 30 min; and 3) 85°C for 5 min, followed by incubation at 4°C.

The reaction mixture for real-time PCR consisted of 4 μL of template cDNA, 10 μL of TaqMan Fast Universal PCR Master Mix (Applied Biosystems), and 1 μL of 20 × TaqMan primers and probe mixture (Applied Biosystems) in a total reaction volume of 20 μL. Using a 7500 Fast Real-time PCR System (Applied Biosystems), real-time PCR was performed with precycling heat activation at 95°C for 20 s, followed by 40 cycles of denaturation at 95°C for 3 s and annealing/extension at 60°C for 30 s. For all of these miRNAs, we used the commercially available TaqMan MicroRNA Assay (Applied Biosystems). The relative quantifications of the candidate miRNAs were analyzed using U6 snRNA (U6) as an internal control as described in our previous study [19].

### Total RNA extraction from FFPE tissue

For the purpose of this study, a 4-μm thick FFPE section stained with hematoxylin and eosin (H&E) was prepared from each block. All H&E slides were reviewed by an expert pathologist, and the suitable blocks for evaluation were selected. From the selected blocks, 50-μm slices were obtained. In each section, approximately 5-mm square (25 mm^2^) slice containing the mucosal surface of the tumor were obtained by use of the technique of macro-dissection, and collected into 1.5-ml tubes. Total RNA was extracted using the miRNeasy FFPE Kit (Qiagen) according to the manufacturer's instructions. Initially, 160 μL of deparaffinization solution (Qiagen) was added to each sample. Finally, total RNA was extracted in 30 μL of RNase-free water. RNA was stored at −80°C until use. RNA concentration and quality were measured according to the method for frozen tissues.

### PTEN expression analysis by real-time RT-PCR

Total RNA extracted from the cancer portions of the frozen tissues from the training cohort patients were analyzed. cDNA was synthesized using a High-Capacity cDNA Reverse Transcription Kit (Applied Biosystems) in accordance with the manufacturer's instructions. The reaction mixture consisted of 30 ng of total RNA, 2 μL of 10 × RT buffer, 2 μL of 10 × Random primer, 0.8 μL of 25 × dNTPs, 1 μL of RNase inhibitor, and 1 μL of MultiScribe reverse transcriptase in a final reaction volume of 10 μL. The thermal cycling conditions were as follows: 1) 25°C for 10 min; 2) 37°C for 120 min; and 3) 85°C for 5 sec, followed by incubation at 4°C. Real-time PCR was performed by same method of miRNA using the commercially available TaqMan Assay (Applied Biosystems, ID: Hs02621230_s1).

### Statistical analysis

In the preliminary study, differences in miRNA expression data were analyzed by t-test. The analyses in the training and validation cohorts were performed as subsequently described. miRNA expression data were analyzed using the comparative cycle threshold (Ct) method. In this analysis, the formula for the relative quantification (RQ) of each gene was calculated using the following formula: (dCt of each miRNA) = (Ct of each miRNA) − (Ct of each internal control), and (RQ of each miRNA) = 2^−(dCtof each miRNA)^. U6 was used as an internal miRNA control in this study [19]. The difference between the two groups was analyzed by the Mann−Whitney *U* test or the chi-square test where appropriate. Logistic regression analysis was used to predict the factors influencing recurrence. Receiver operating characteristic (ROC) curves with the Youden index were established for determining the threshold of RQ in miRNAs to differentiate between patients with or without recurrence. In addition, the area under the curve (AUC) was established using the ROC curve. The Spearman correlation test was used to examine correlation between miRNA expression in matched frozen and FFPE CRC tissues. Relapse-free survival (RFS) curves were analyzed using the Kaplan–Meier method, and differences were examined using log-rank tests. Cox regression analyses were performed to evaluate the independent predictive factors of RFS.

In the preliminary study, statistical analyses were performed using Cluster 3.0 software (available at http://bonsai.hgc.jp/~mdehoon/software/cluster/). The results were visualized by Java TreeView (available at http://jtreeview.sourceforge.net/). In the training and validation studies, statistical analyses were performed using IBM SPSS Statistics version 20 (IBM, Armonk, NY). A *P* value of < 0.05 was considered statistically significant.

## SUPPLEMENTARY MATERIALS FIGURES AND TABLES


