# Supplementary material for: High expression of miR-181c as a predictive marker of recurrence in stage II colorectal cancer
Source: Oncotarget. 2016 Dec 28;8(4):6970–83. doi: 10.18632/oncotarget.14344 (PMC5351683; doi:10.18632/oncotarget.14344)
Supplement: Supplementary file 1 [file oncotarget-08-6970-s001.pdf]

High expression of miR-181c as a predictive marker of recurrence in stage II colorectal cancer

SUPPLEMENTARY FIGURES AND TABLE

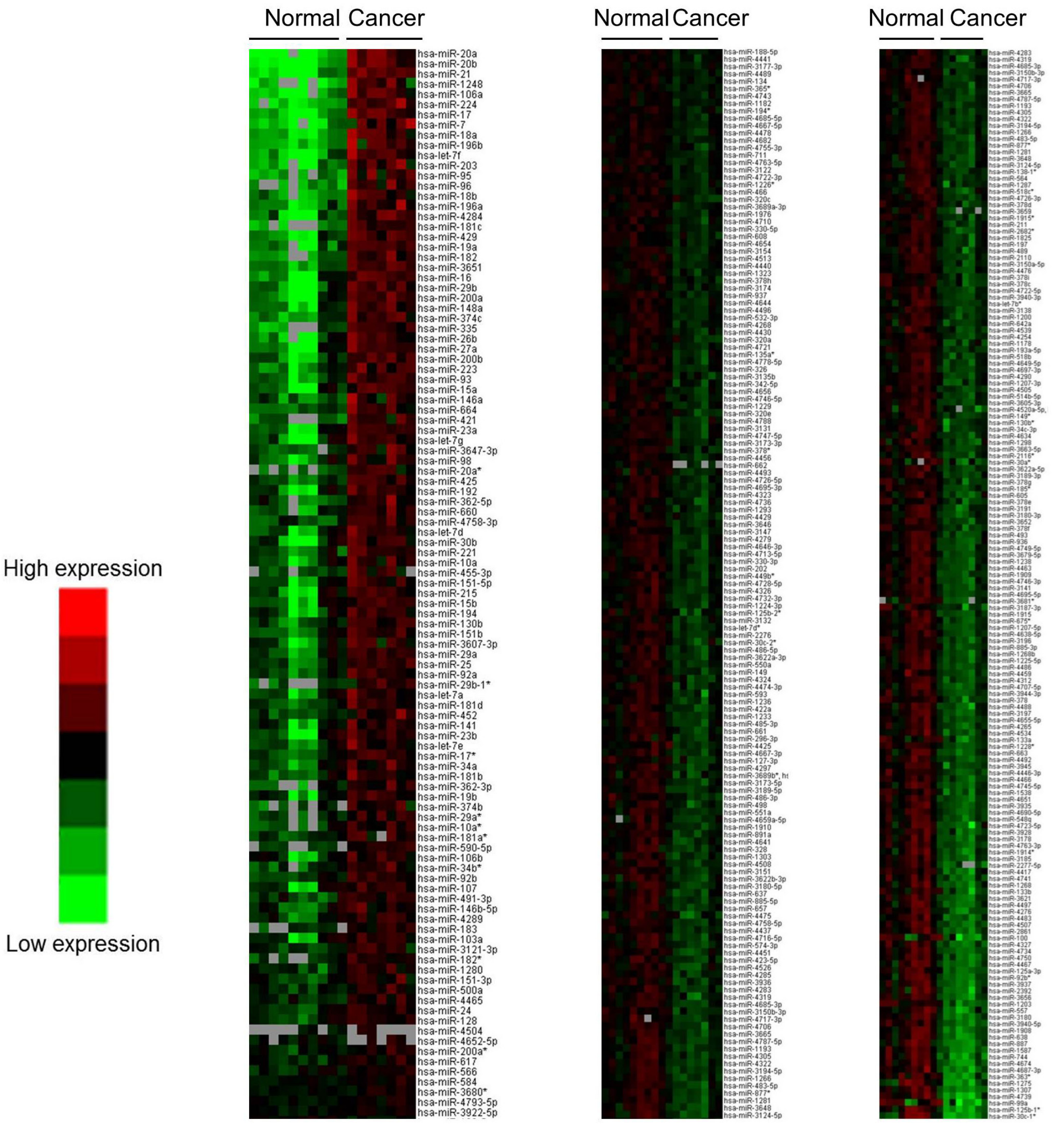

Supplementary Figure 1: Differences in the miRNA expression in the frozen tissues between cancerous and noncancerous tissues using a heat map for the preliminary study. Totally, 394 miRNAs showed different miRNA expressions between cancerous and noncancerous tissues ($P < 0.01$). In the 394 miRNAs, 105 miRNAs showed higher expressions in the cancer tissues than in the normal tissues.

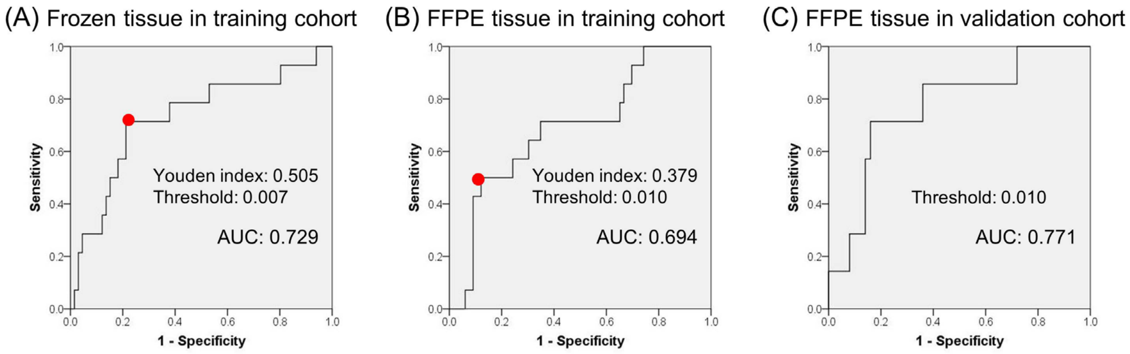

Supplementary Figure 2: ROC curve of miR-181c for recurrence. A. Frozen tissues in the training cohort. **B.** FFPE tissues in the training cohort. **C.** FFPE tissues in the validation cohort.

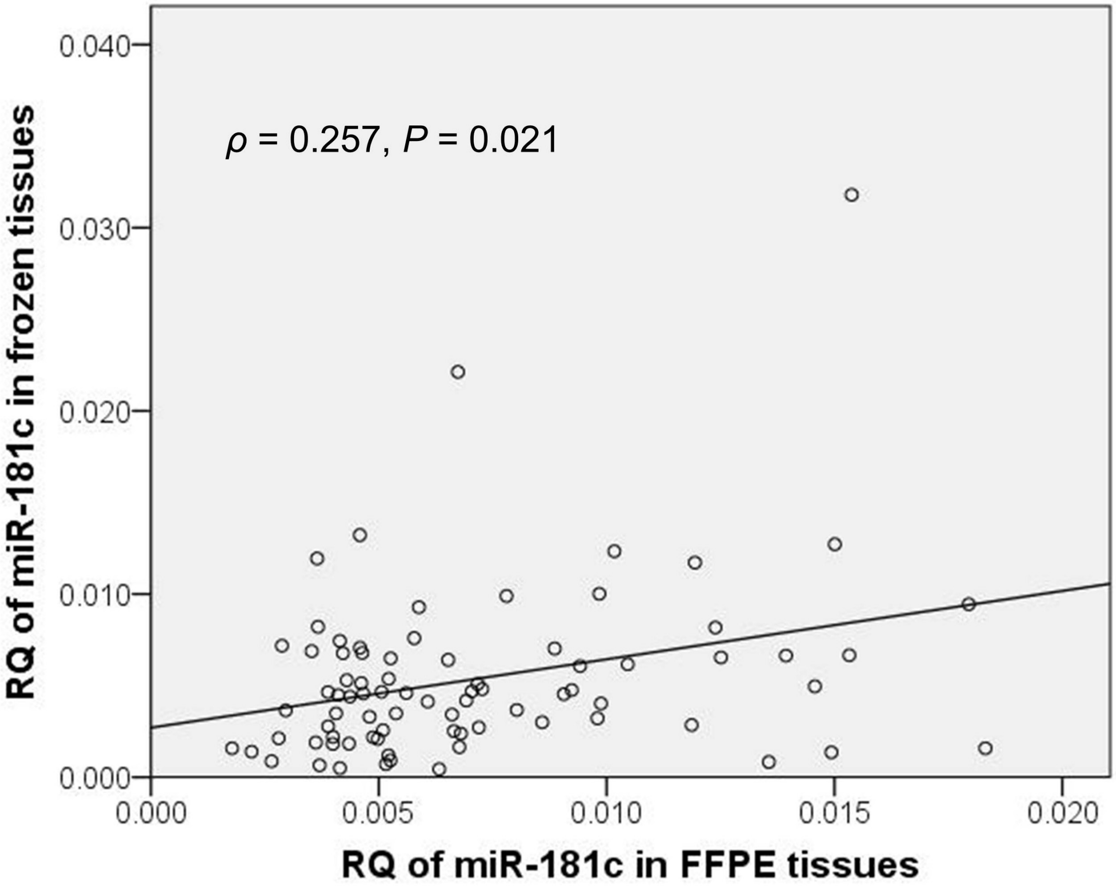

Supplementary Figure 3: Scatter plots showing the correlation between relative quantification of miR-181c in frozen and matched FFPE tissues obtained from 80 CRC patients in the training cohort. A positive correlation was found by Spearman correlation ($\rho = 0.257$, $P = 0.021$).

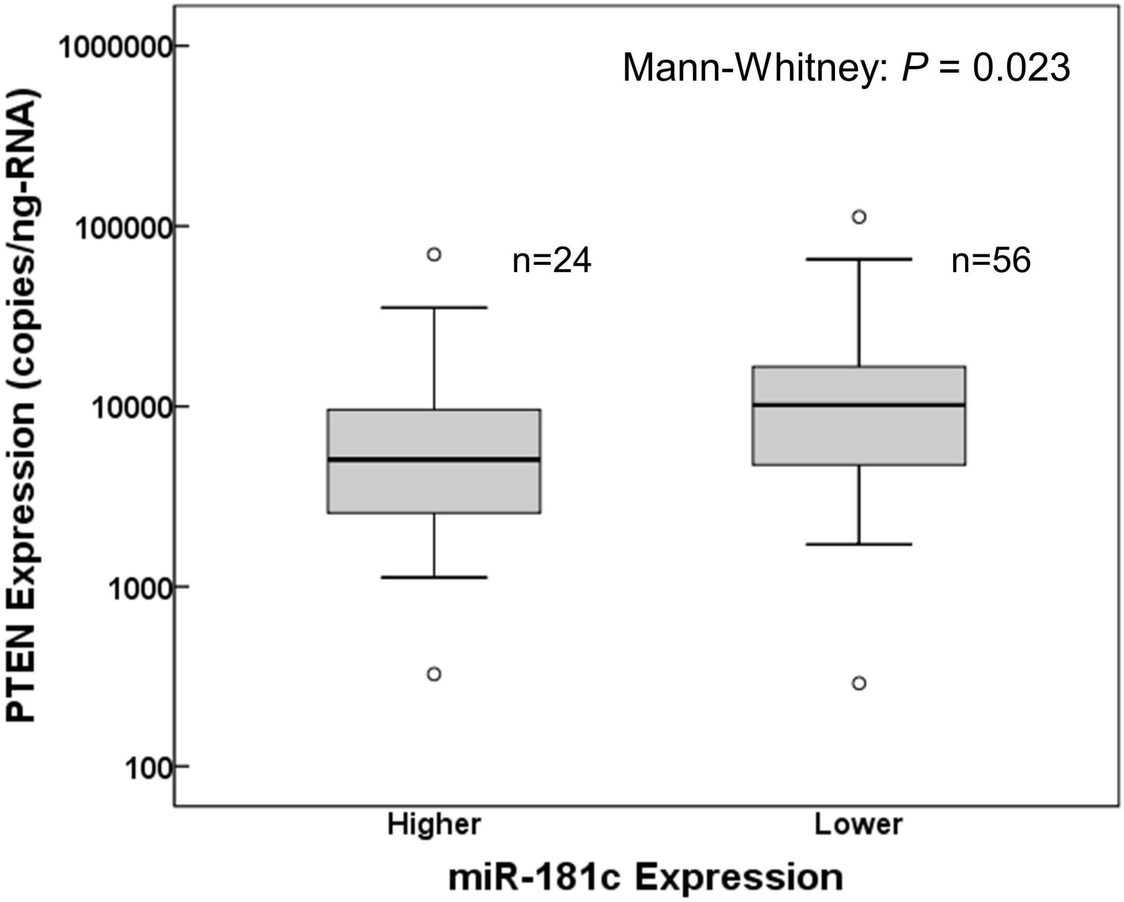

Supplementary Figure 4: The correlation between the expression of miR-181c and PTEN in training cohort using frozen tissues. The expression of PTEN was significantly lower in the patients with higher miR-181c expression than those with lower expression ($P = 0.023$).

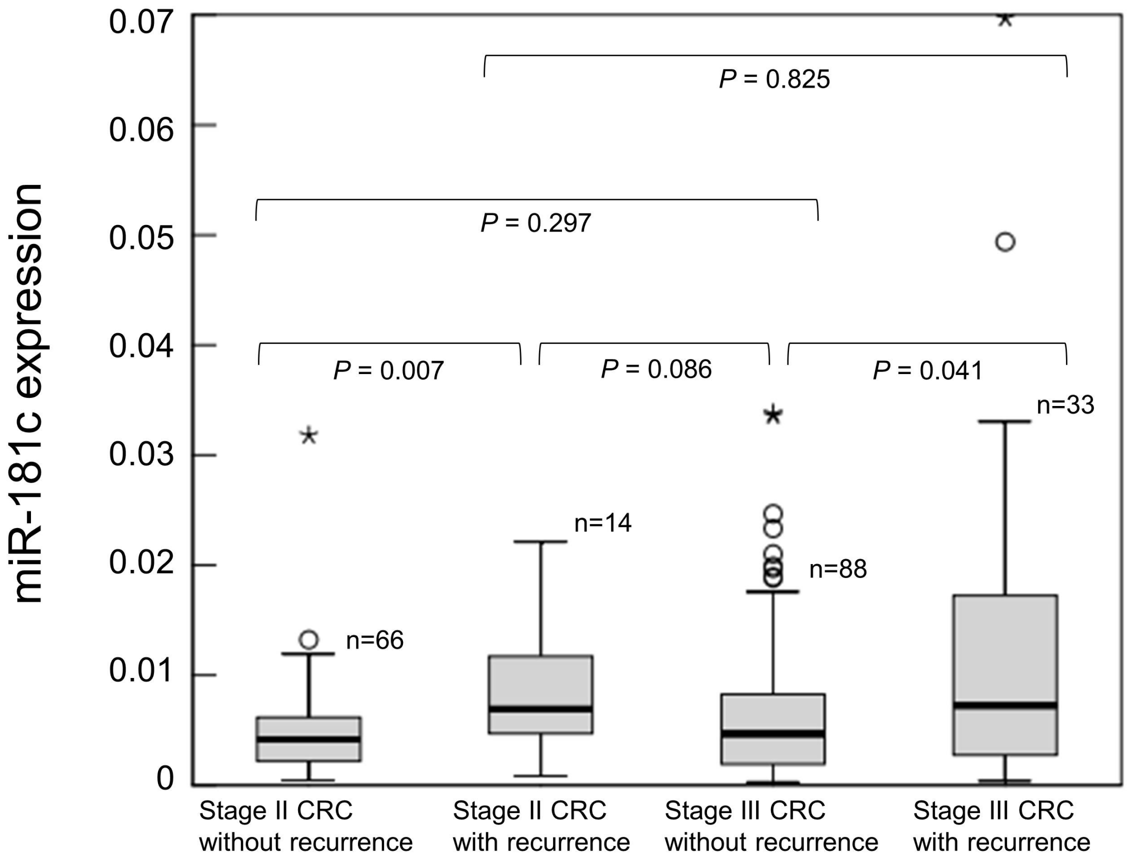

Supplementary Figure 5: The expression of miR-181c compared with stage II CRC patients and stage III CRC patients in the preliminary study using frozen tissues. The expression of miR-181c in stage II CRC patients without recurrence was not showed significantly difference from that in the stage III CRC patients without recurrence ($P = 0.297$). Moreover, that in stage II CRC patients with recurrence was similar compared with that in stage III patients with recurrence ($P = 0.825$). In addition, that in stage III CRC patients with recurrence was significantly higher with that in stage III patients without recurrence ($P = 0.048$).

Supplementary Table 1: Ct value of miRNA in the frozen tissue and FFPE tissue of training and validation cohort

	Training cohort				Validation cohort			
	CRC patients with recurrence, n=14		CRC patients without recurrence, n=66		CRC patients with recurrence, n=7		CRC patients without recurrence, n=50	
	median	range	median	range	median	range	median	range
Frozen Tissue								
let-7a	23.7	21.6—26.2	24.8	21.9—28.8				
let-7d	26.4	25.0—28.1	27.1	24.4—31.7				
let-7e	23.1	21.0—24.7	23.8	20.6—26.4				
miR-18b	36.8	31.4—39.8	37.4	33.1—39.9				
miR-23c	30.0	28.2—34.3	31.2	27.7—36.1				
miR-26b	23.3	21.8—24.0	23.5	21.8—25.9				
miR-128a	26.8	26.4—29.0	27.6	26.1—30.0				
miR-146b	23.2	22.4—25.0	23.8	21.3—25.1				
miR-148b	26.2	27.3—29.3	28.2	26.9—30.7				
miR-151-5p	26.6	24.8—28.1	27.3	25.0—28.1				
miR-181c	28.6	27.4—30.2	29.5	27.2—32.2				
miR-221	23.7	22.7—25.7	24.1	22.3—25.8				
miR-222	22.6	22.0—24.0	22.7	21.0—23.8				
miR-361	27.2	26.5—29.4	28.4	26.5—31.0				
miR-500	29.1	27.7—31.1	29.5	28.0—31.5				
miR-24	20.7	19.8—21.7	21.1	19.4—22.8				
RNU6B	27.7	25.2—30.7	28.0	24.7—33.0				
U6snRNA	21.3	18.2—23.3	21.4	18.1—23.0				
FFPE tissue								
let-7a	23.2	21.3—23.8	23.0	21.4—25.3				
let-7d	24.2	23.2—24.8	24.2	23.5—26.1				
let-7e	22.5	21.4—23.4	22.6	21.5—24.4				
miR-23c	27.1	25.7—28.8	27.0	26.0—28.5				
miR-26b	23.1	21.8—23.9	23.3	22.1—25.3				
miR-128a	27.4	26.7—28.3	27.7	26.4—28.9				
miR-148b	27.1	26.0—27.7	27.4	26.1—28.4				
miR-151-5p	26.3	25.2—27.6	26.5	25.0—35.3				
miR-181c	27.8	26.3—28.9	28.0	26.5—30.0	28.2	27.1—29.5	29.3	27.5—32.0
miR-361	27.8	26.4—28.8	27.8	26.2—29.7				
U6snRNA	20.6	20.1—21.8	20.7	19.4—22.5	21.8	20.5—22.6	21.4	19.8—23.9

CRC: colorectal cancer.
